# The Modern Treatment of Uterine Fibroids

**Published:** 1900-05-05

**Authors:** 


### THE MODERN TREATMENT OF UTERINE FIBROIDS.

In a clinical lecture at the Middlesex Hospital Dr. William Duncan recently discussed the proper treatment of uterine fibroids in the light of modern experience. After pointing out how considerable is the danger which the presence of this condition involves, more especially in cases in which the patient may become pregnant, he came to the question of treatment, which practically is the question of surgical treatment, although in some cases the administration of ergot may do some good in the prevention of hæmorrhage. Surgical treatment may take the form of curettage of the endometrium, removal of the ovaries and tubes, vaginal enucleation or morcellement, myomectomy, and hysterectomy.

**Curettage.**—This not infrequently gives great, if only temporary, relief, and it is a wise procedure always to explore the uterine cavity before recommending hysterectomy or other severe operation for uterine hæmorrhage, as sometimes polypi or small submucous myomata may be found and readily removed, with complete relief, even though a large tumour still remains in the uterine wall.

**Removal of the Ovaries and Tubes.**—This is an operation which has now rightly fallen into disuetude. The mortality from it was not insignificant, the appendages in some cases could not be removed, and the operation sometimes failed to produce the desired effect.

**Vaginal Enucleation.**—Although this operation has some advocates at the present day, Dr. Duncan considers it a most unsurgical and dangerous method of treating submucous fibroids unless these be quite small and in process of becoming polypoid. In such cases as the latter it has its value, but he feels confident that in the vast majority of cases it has a higher mortality than hysterectomy and no compensating advantages; for even the saving of the uterus is but a disadvantage and a source of possible future danger. No one, he says, could avoid contemplating with dread the possibility of a woman becoming pregnant who had had a myoma enucleated from her uterus.

**Myomectomy.**—This is indicated where the tumour is sub-peritoneal and attached to the uterus by a pedicle. But even this operation is not so safe as hysterectomy, and, moreover, it leaves the danger of a damaged uterus.

**Hysterectomy.**—Of the various proceedings for the removal of the uterus, Dr. Duncan expresses a very decided preference for the intra-peritoneal operation, in which a small piece of the cervix is left so as to avoid opening the vagina. With regard to extra-peritoneal hysterectomy, in which the stump of the uterus left is clamped by a serre nœud, brought outside the abdomen, and fixed at the lower corner of the external incision, he regards it as "a relic of barbarism," and says that "any surgeon who practises it fails in his duty to his patients by not keeping abreast of the advance of surgical science." In doing the operation which he recommends, Dr. Duncan advises that, if possible, the ovary and tube on one side should be left, by which means the discomforts of a premature menopause are avoided; he ties the uterine vessels, as well as the ovarian, before cutting the peritoneal flaps, as the patient is in this way spared some hæmorrhage; and he does not trouble to close the upper end of the cervical canal. In regard to mortality, he gives a table of 68 cases in which he has done intra-peritoneal hysterectomy with four deaths, but one of these deaths occurred when the patient was convalescent and about to leave the hospital, so that he places his mortality at four and a-half per cent.; but he thinks that where the operation is an uncomplicated one the mortality should not be more than one or two per cent.

